# Perinephric urinoma following spontaneous renal rupture in the third trimester of pregnancy: a case report and brief review of the literature

**DOI:** 10.1186/s12884-019-2669-9

**Published:** 2019-12-18

**Authors:** Ya Chen, Yun Fang Yan, Ying Zhang, Xianming Carroll, Hui Rong Li, Li Tao, Mei Guo Sun, Sandra Leeper-Woodford

**Affiliations:** 1 Department of Obstetrics and Gynecologys, the First Affiliated Hospital of Anhui Medical University, Hefei, Anhui Province China; 2 Anhui Province Key Laboratory of Reproductive Health and Genetics, Biopreservation and Artificial Organs, Anhui Provincial Engineering Research Center, Hefei, Anhui Province, China; 3 Anhui Medical University, Hefei, Anhui Province China; 4 Department of Public Health, Mercer University College of Health Professions, Atlanta, Georgia USA; 5 Department of Biomedical Sciences, Mercer University School of Medicine, Macon, Georgia USA

**Keywords:** Spontaneous urinoma, Renal rupture, Pregnancy

## Abstract

**Background:**

Spontaneous formation of urinoma is a rare condition, especially for pregnant women. We report a patient in the third trimester of pregnancy with a spontaneous renal rupture who then develops a urinoma from urine leaking into the perinephric space.

**Case presentation:**

A 23-year-old primagravida was diagnosed with a spontaneous renal rupture and acute left loin pain accompanied by hematuria when she was 35 weeks pregnant. A sub-capsular perinephric cyst then developed to a size of 319 × 175 × 253 mm, and because of discomfort to the patient, we performed Cesarean section. After a healthy male newborn was delivered, fluid was suctioned from a large perirenal cyst that had an estimated size of 300 × 200 × 300 mm. A percutaneous nephrostomy tube was left in the cyst until CT showed no remaining fluid. In the six-month follow-up, the patient showed no perirenal extravasation according to an ultrasound scan, and the urine analysis and renal function tests were normal.

**Conclusion:**

Close follow-up should be recommended for the patient who has renal rupture after conservative therapy, especially for pregnant woman. CT or MRI should be considered in addition to utilizing ultrasound in the management of pregnant women who present with urinomas. Percutaneous nephrostomy is suggested as an appropriate treatment for large urinomas.

## Background

Urinoma or perirenal pseudocyst is defined as extravasation of urine into the retroperitoneal space [1]. The extravasated urine will induce tissue inflammation and fibrosis, which will result in formation of an encapsulated sac surrounding the aggregated urine [2]. The major causes of the urine extravasation are external trauma and urinary tract obstruction [3, 4]. Spontaneous formation of a perinephric urinoma is very rare, especially for pregnant women [1, 5–7]. We report a pregnant woman who developed a large urinoma following spontaneous renal rupture, without any traumatic factors, during her third trimester of pregnancy. We will also further review the literature related to this issue in pregnancy.

## Case presentation

**Fig. 1:**
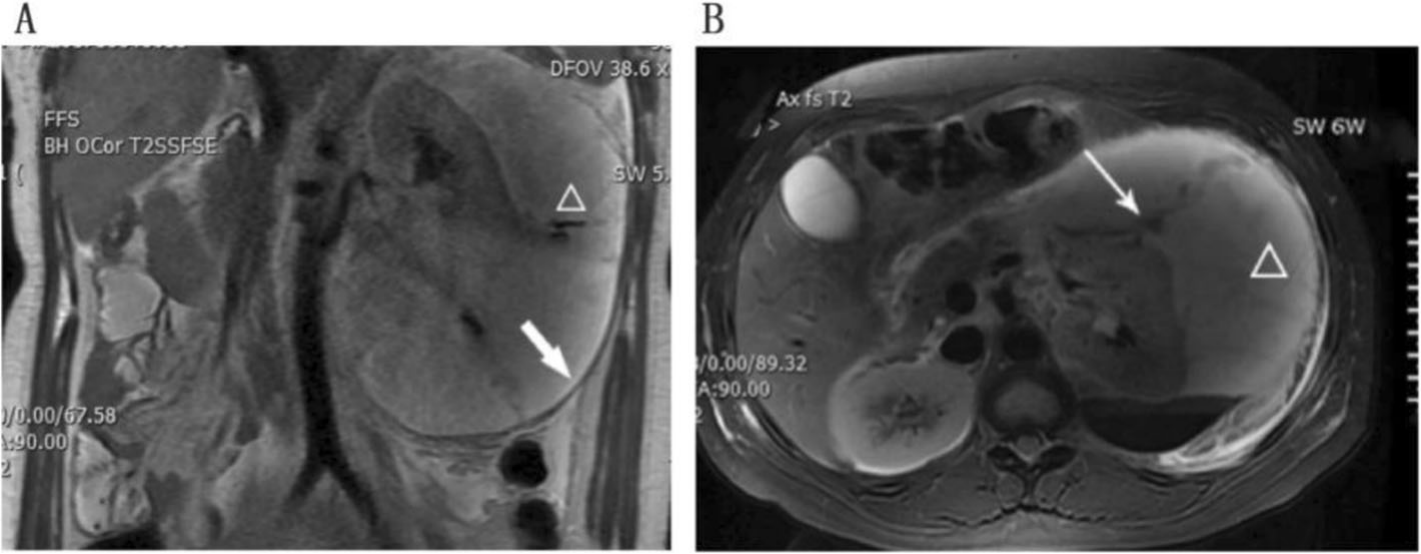
**a** Coronal slice of MRI on the first visit, the image showed the fluid(△) localized under the left renal capsule (→). **b** Axial image of MRI on the first visit showed the rupture of her left kidney (→) and the fluid extravasation around it (△)

A 23-year-old, nulliparous, Chinese woman at 35 weeks gestation, was admitted to the hospital complaining of hematuria accompanied by severe pain in the left abdomen and left subcostal area. She had no past history of urological difficulties until week 26 of pregnancy when she was diagnosed with a spontaneous renal rupture resulting in acute left loin pain and hematuria. She denied any accident or renal problems at that time. Magnetic resonance imaging (MRI) confirmed the diagnosis (Fig. 1a, b), and that the fluid around the ruptured left kidney had spread from the superior margin of the 10th thoracic vertebral body to the inferior margin of the second lumbar vertebral body. The patient was admitted to the hospital for observation. The hematuria resolved the day following admission, and she was discharged 1 week after admission when the loin pain decreased and her hemodynamic status was stabilized.

At 34 weeks gestation, she presented at the urology clinic with a growing mass in the left subcostal area. Ultrasound showed right renal hydronephrosis without hydroureter. In addition, a cyst was noted to surround the left kidney, and this was measured as 250 × 170 × 233 mm. The cyst was located under the left renal capsule, and the margins were 100 mm from the upper pole, and 60 mm from the lower pole of the left kidney. The medial margin of the cyst was 70 mm away from the medial edge of the left kidney, and 103 mm away from the lateral edge. It was suspected that she had a subcapsular hematoma following renal rupture. At the time, she was more than 33 weeks pregnant, and her laboratory values were normal for kidney function, hematocrit and hemoglobin. She declined further testing of the mass because she was concerned about the side effects on the fetus.

The patient came to the Obstetric Department requesting a cesarean section when she was 35 weeks pregnant because she could not bear the severe pain of the increasingly swollen left subcostal mass. She denied nausea, shortness of breath, fever or chills, and there was no vaginal bleeding or uterine contractions. After she was admitted to the obstetrics ward, the fetal heart non-stress test was determined to be reactive. The fetal bi-parietal diameter (BPD) measured by ultrasound was 86 mm, and femoral length. (FL) was 66 mm, which were both consistent with the gestational age. The amniotic fluid index was 95 mm and umbilical arterial S/D was 2.5. The biophysical profile score was 8. Additionally, ultrasound demonstrated signs of maternal hydronephrosis accompanied by a much larger cyst surrounding the left kidney than that observed in the previous ultrasound. Over the past few weeks, this sub-capsular cyst had increased to a size of 319 × 175 × 250 mm.

Physical examination revealed the gravid uterus, and obvious swelling over the left abdominal region. A large firm mass with undefined boundary was palpable over the left lumbar region extending to the side of uterus. The left flank and subcostal area had notable tenderness with significant hyperesthesia of the overlying skin. Laboratory evaluation demonstrated a normal white blood cell count and hemoglobin. The urinalysis and kidney function values showed no abnormalities.

The patient was known to have had a spontaneous renal rupture and a growing mass in the left subcostal area at week 26 of gestation. The first diagnosis was hematoma or abscess surrounding the left kidney. Because her hemodynamic status was stable without anemia or fever for more than 2 months after the renal rupture, she had been followed using repeat urine analyses, which showed no hematuria. The urologist diagnosed her current condition as perirenal urine extravasation, and it was decided to place a percutaneous nephrostomy tube (PCN) before delivery or during the surgery.

**Fig. 2:**
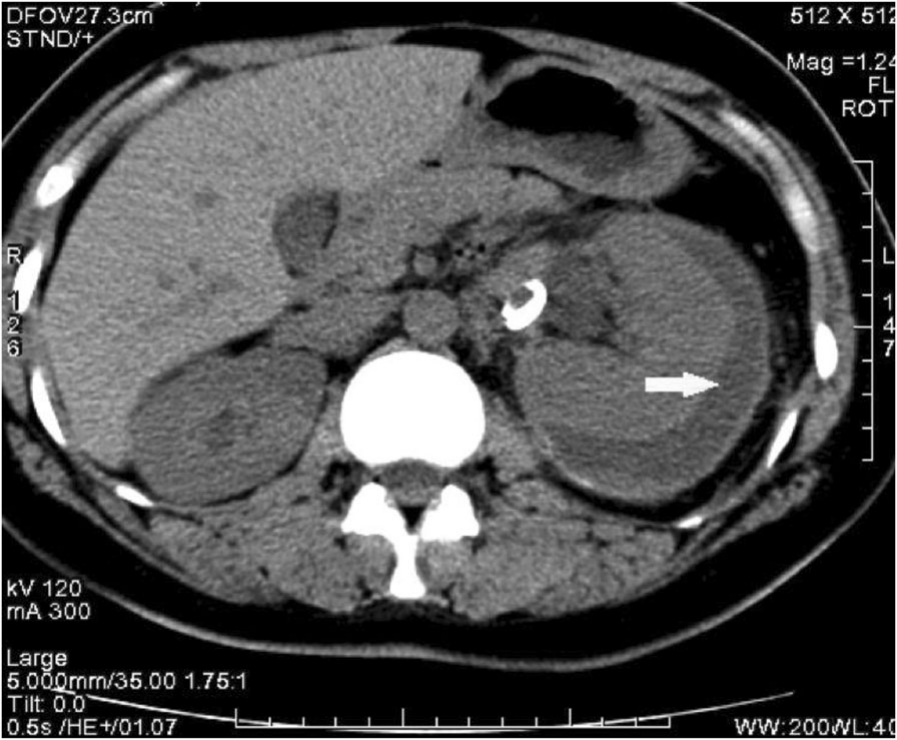
Two month after surgery the Coronal slice CT scan image showed the sub-capsular fluid (→)

The patient requested Cesarean section because the gestational age was 35 weeks, and due to concerns about the progression of the subcostal mass, the surgery was performed. The patient agreed to receive the PCN placement along with the Cesarean section. A male newborn weighing 2580 g with an Apgar score 10/10 at 1 and 5 min was delivered. Following completion of the Cesarean section, the patient’s upper abdominal cavity was explored, and, on the left side, a large retroperitoneal bulging mass was noted with an estimated size of 300 × 200 × 300 mm. The consulting urologist suctioned 5 ml of light yellow fluid from the mass and the sample was sent for creatinine determination. The creatinine level of the sample was 2100 μmol/L and similar to the level in the patient’s urine. A left urinoma as a result of spontaneous renal rupture was then diagnosed, and a total of 4 L of fluid was drained via the PCN. PCN was left in the urinoma to allow for further drainage of urine. After 3 days, a computer tomography (CT) scan was performed to assess the effect of draining the urinoma. The CT revealed that the PCN was properly placed in the cyst, and that the urinoma had decreased in size. The patient was discharged 1 week after the surgery with the PCN in place. She was closely followed by a urologist, and the nephrostomy tube was to be removed when there was no further drainage. Two months postpartum, the CT scan showed the cyst had diminished in size to 50 mm (Fig. 2). Six months later, an ultrasound scan was performed and revealed that no perirenal extravasation was present. The urine analysis and renal function tests at that time were normal.

## Discussion and conclusion

Urinoma is an acute complication that occurs following an injury to the kidney or upper urinary tract. Urine extravasation as a result of renal trauma is common, but development of urinoma may only occur in a few cases [1]. Urine may leak into the perirenal tissues resulting in liquefaction and formation of fibrous tissues which forms the perirenal pseudocyst [8]. The conditions for formation of urinoma include impairment of the renal collecting system, urinary extravasation, and ureteral obstruction [9, 10].

Spontaneous extravasation of urine to the perinephric space and development of a maternal urinoma is an uncommon complication during pregnancy [5–7, 11]. Common etiological factors for urinoma development in pregnant women include renal injury or urinary tract obstruction are [12]. In pregnant women, cases of spontaneous rupture of the kidneys and renal tract rupture have been reported, but development of urinomas as a result of these is very rare [13, 14].

Renal rupture may occur in the kidney parenchyma or in the renal collecting system. Spontaneous or traumatic rupture of the kidney may occur, primarily in kidneys with underlying anatomic abnormalities since those kidneys are susceptible to minimal traumatic insults [15–17]. Patients who have renal tract ruptures have been treated with double-J tube placements, which can provide sufficient drainage of the urine [18, 19]. During pregnancy, common causes of renal parenchymal rupture are renal aneurysms and trauma. Tubular calculi and obstruction of the lower urinary tract are common causes of rupture of the renal collecting system [12]. In patients with renal parenchymal rupture, the main concern is to stabilize hemodynamic status in the patient because both the mother and fetus may be in danger [20].

An important cause of temporary urinary tract obstruction during pregnancy may be due to increased uterine compression or increased ureteral pressure. This condition may occur when renal pelvis pressure exceeds a critical level between 70 and 75 mmHg due to ureteral or renal compression [21]. Urinoma in the left flank is more rare than in the right, because the uterus exerts greater pressure on the right ureter unless the gravid uterus is rotated more towards the left side [22, 23]. In the current patient, a spontaneous left renal parenchymal rupture occurred following a period of observation during her third trimester of pregnancy when the pressure increased within the urinary system and caused urine leakage into the perinephric space.

Diagnosis of this condition depends primarily on imaging studies with ultrasound used as the primary test [3, 24]. Abnormal ultrasound images may include: 1) discontinuity of the renal parenchymal or urinary tract; 2) fluid and dark areas surrounding the affected kidney; 3) a section of the ruptured kidney capsule floating in the surrounding fluid areas. In spite of its usefulness, the ultrasound has some limits on detecting or locating small ruptures. Following the abnormal findings using ultrasound, further tests including, CT, MRI or intravenous urography (IVU) are also recommended [25, 26]. Compared with the results using ultrasound, these other methods can be used to locate the rupture site, and more accurately estimate the size of the urinoma. These other methods can also delineate more clearly the relationship between the urinoma and surrounding tissues. Utilizing these methods facilitates making the diagnosis and planning the treatment protocol. Misdiagnosis of a urinoma could delay treatment and healing of the renal rupture, which may lead to development of severe complications. Hypertension, urinary peritonitis, renal atrophy and kidney failure are possible in patients with misdiagnosed urinomas [27, 28]. For this reason, awareness and monitoring of this condition during pregnancy should be noted.

In conclusion, pregnant women who experience renal rupture, an ultrasound examination should be administered every two to 4 weeks following the initial examination that reveals possible rupture, or renal contusion and laceration. In patients who develop a perinephric cyst, assessment of the kidney on the affected side should be carefully monitored. If a urinoma occurs in these patients, double-J tube insertion is suggested as the initial treatment prior to any other treatment interventions. This tube insertion into the urinoma will provide better and faster drainage of the fluid, and reduce pressure on the kidney. Following stabilization of the hemodynamics of the patient, a period of percutaneous drainage with a nephrostomy tube is then recommended after initial treatment of the urinoma.

## Data Availability

The datasets analyzed and the materials used during the current report are available from the corresponding author on reasonable request.

## Abbreviations

BPD: Biparietal diameter
CT: Computed tomography
FL: Femoral length
IVU: Intravenous urography
MRI: Magnetic resonance imaging
PCN: Percutaneous nephrostomy tube

## References

[1] Ushioda N, Matsuo K, Nagamatsu M, Kimura T, Shimuya K (2008). Maternal urinoma during pregnancy. J Obstet Gynaecol Res.

[2] Goldwasser J, Wahdat R, Espinosa J, Lucerna A (2018). Urinoma: prompt diagnosis and treatment can prevent abscess formation, Hydronephrosis, and a progressive loss of renal function. Case Rep Emerg Med.

[3] Lee J, Darcy M (2011). Renal cysts and Urinomas. Semin Interv Radiol.

[4] Hudson H, Hundley R (1972). Pararenal Pseudocyst. Br J Urol.

[5] Hamoud K, Kaneti J, Smailowitz Z, Kroll D, Barki Y (1994). Spontaneous Perinephric Urinoma in pregnancy. Int Urol Nephrol.

[6] Middleton AW, Middleton GW, Dean LK (1980). Spontaneous renal rupture in pregnancy. Urology..

[7] Noe HN, Raghavaiah NV (1980). Spontaneous peripelvic extravasation of urine during pregnancy. South Med J.

[8] Trehan A, Takhtani D, Singh S, Kumar L (1998). Urinoma-AnUnusual complication following kidney biopsy. Indian J Pediatr.

[9] Koelmeyer TD, Ferguson RS, Nicholls SC (1977). Pararenal Pseudocyst. J Trauma.

[10] Chang H, Kuei C, Tseng C, Hou Y, Tseng Y (2018). Spontaneous perirenal urinoma induced by NSAID-associated acute interstitial nephritis. Ther Clin Risk Manag.

[11] Maresca L, Koucky CJ (1981). Spontaneous rupture of the renal pelvis during pregnancypresenting as acute abdomen. Obstet Gynecol.

[12] Van Winter JT, Ogburn PL, Engen DE, Webb MJ (1991). Spontaneous renal rupture during pregnancy. Mayo Clin Proc.

[13] De Wilde R, Raas P, Hesseling M (1988). Spontaneous rupture of the kidney pelvis in pregnancy. Geburtshilfe Frauenheilkd.

[14] Joechim GR, Becker EL (1965). Spontaneous rupture of the kidney. Arch Intern Med.

[15] Wang C, Li X, Peng L, Gou X, Fan J (2018). An update on recent developments in rupture of renal angiomyolipoma. Medicine..

[16] Hellmund A, Meyer C, Fingerhut D, Müller SC, Merz WM, Gembruch U (2016). Rupture of renal artery aneurysm during late pregnancy: clinical features and diagnosis. Arch Gynecol Obstet.

[17] Waltzer WC (1981). The urinary tract in pregnancy. J Urol.

[18] Satoh S, Okuma A, Fujita Y, Tamaka M, Nakano H (2002). Spontaneous rupture of the renal pelvis during pregnancy: ACase report and review of the literature. Am J Perinatol.

[19] Matsubara S, Morita T, Saito Y, Sato S, Suzuki M (2010). Non-traumatic rupture of the left upper urinary tract during pregnancy without discernable underlying disorders. Arc Gynecol Obstetr.

[20] Pontis A, Piras B, Meloni A, Lisa AD, Melis GB, Angioni S (2013). Rupture of renal angiomyolipoma in pregnancy. J Obstet Gynaecol.

[21] Rubi RA, Sala NL (1968). Ureteral function in pregnant women. 3. Effect of different positions and of fetal delivery upon ureteral tonus. Am J Obstet Gynecol.

[22] Cheung KL, Lafayette RA (2013). Renal physiology of pregnancy. Adv Chronic Kidney Dis.

[23] Samir N (1985). Beydoun. Morphologic changes in the renal tract in pregnancy. Clin Obstet Gynecol.

[24] Christopher T, Matthew E, James M (2018). Point-of-care ultrasound identifies Urinoma complicating simple renal colic: a case series and literature review. J Emerg Med.

[25] Yang DM, Jung DH, Kim H, Kang JH, Kim SH, Kim JH, Hwang HY (2004). Retroperitoneal cystic masses: CT, clinical, and pathologic findings and literature review. Radiographics..

[26] Titton RL, Gervais DA, Hahn PF, Harisinghani MG, Arellano RS, Mueller PR (2003). Urine leaks and Urinomas: diagnosis and imaging-guided intervention. Radiogr Rev Publication Radiol Soc North America Inc.

[27] Jalbani I, Ather M (2014). Renal Forniceal rupture in pregnancy secondary to obstructive renal stone presenting with acute renal failure. Saudi J Kidney Dis Transpl.

[28] Cheng JW, Li A, Chamberlin DA (2018). Perinephric Urinoma secondary to malignancy in a pediatric patient. Urology..

